# A thermo-physiological description of a 50^th ^percentile Western female

**DOI:** 10.1186/2046-7648-4-S1-A138

**Published:** 2015-09-14

**Authors:** Mark Hepokoski, Scott Gibbs, Allen Curran, David Nelson

**Affiliations:** 1 ThermoAnalytics, Calumet, MI, USA; 2 University of South Alabama, Mobile, AL, USA

## Introduction

Thermophysiological models are used to predict thermal sensation, thermal comfort and human effectiveness for a wide range of environmental conditions. Typically, such models are based on the anatomy and physiological responses of an adult male. The objective of this study was to develop an adult female model and test it against experimental results from the literature.

## Methods

A 20-segment, 50^th ^percentile western female model was developed for use with an existing human thermoregulation model [1]. A complete set of system parameters, including passive (height, weight, surface area, basal metabolism and cardiac output) and active (sweating, shivering, vasomotor responses) values, were derived from modern anthropometric and thermophysiologic data in the open literature. Sweating, shivering, and vasomotion were scaled from male values to better reflect female thermoregulatory function. The female model was tested by comparing its results (core temperature, whole body sweat rate, skin blood flow) to published experimental results obtained during exercise in male and female cohort groups [2].

## Results

**Figure 1:**
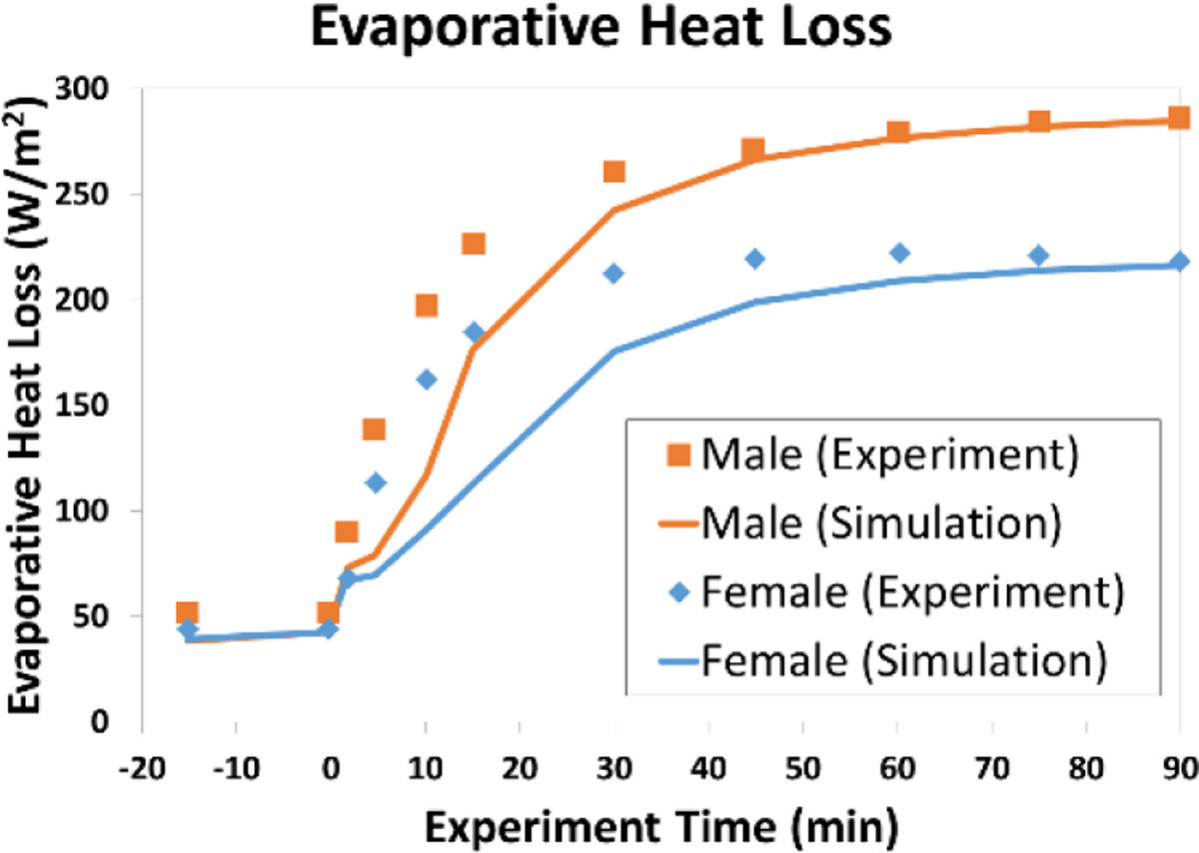
**Evaporative heat loss during exercise at 50% VO_2,max_**.

**Figure 2:**
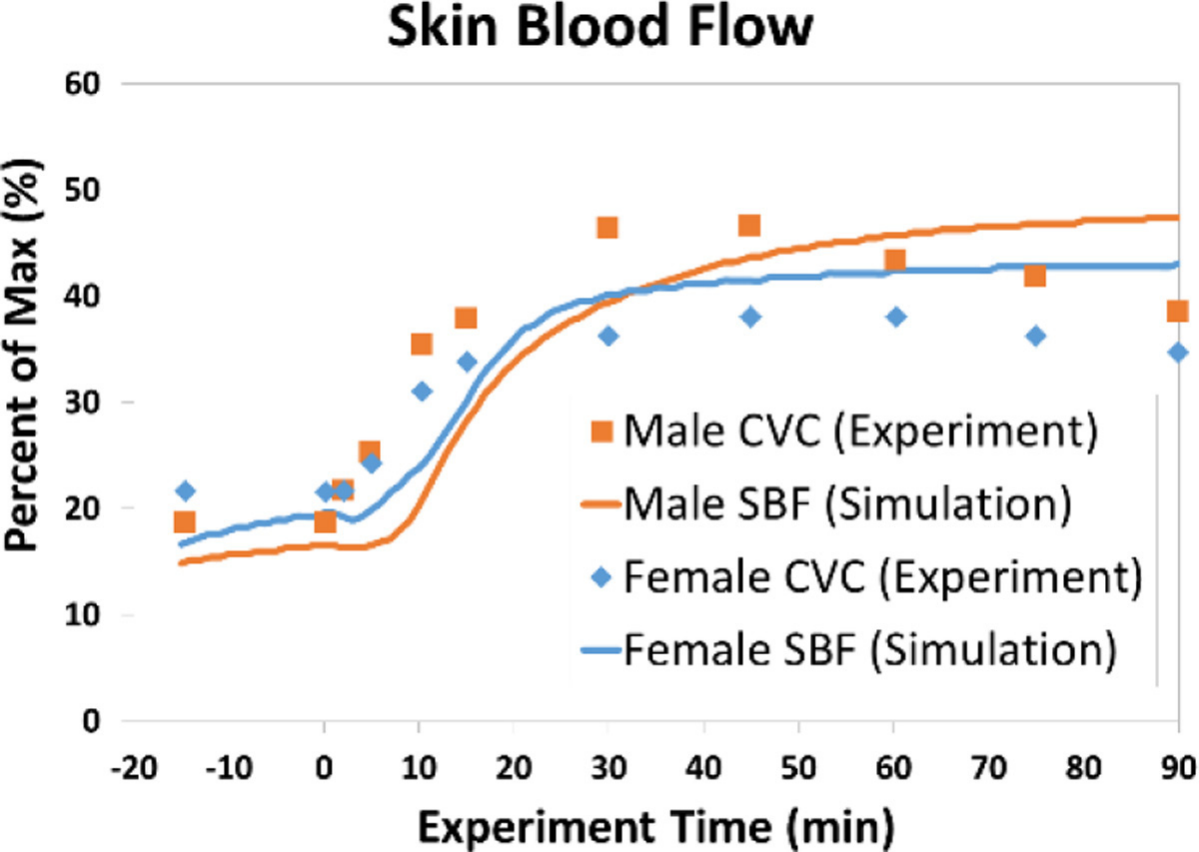
**Skin blood flow responses during exercise at 50% VO_2,max_**.

Predicted and measured evaporative heat losses and rates of skin blood flow during exercise at 50 % VO_2,max _are shown in Figures 1 and 2. Male responses are provided for comparison purposes. The rise in rectal temperature for the female during the exposure was ΔT_re,simulation _= 1.32 °C (ΔT_re,experiment _= 1.22 °C).

## Discussion

The model predictions are generally consistent with experiment. An apparent lag in the onset of thermoregulatory response was observed in the model. This can likely be attributed to the model's lack of a capability to consider physical fitness. Despite this limitation, the female model accurately predicts the lower rate of evaporative heat loss compared to the male. Skin blood flow predictions are in good agreement with the male model and match the trends in the experiment (which deemed differences in observed cutaneous vascular conductance (CVC) between females and males to be negligible [2]).

## Conclusion

A female thermophysiology model developed from anthropometric data is more capable of simulating thermoregulatory response (core temperature, sweat rate and skin blood flow) in exercising women, compared with an existing model based on a standard adult male. Neither model incorporates effects of physical fitness, which may affect thermoregulatory function [3]. Current efforts are aimed at refining the models to reflect the influences of fitness and age on thermoregulation in either gender.
